# Black Soybean Seed Coat Extract Suppresses Gut Tumorigenesis by Augmenting the Production of Gut Microbiota-Derived Short-Chain Fatty Acids

**DOI:** 10.3390/cancers16223846

**Published:** 2024-11-15

**Authors:** Yasuyuki Shimizu, Shunta Hirano, Mohammed Salah, Namiko Hoshi, Yoko Yamashita, Takeshi Fukumoto, Naritoshi Mukumoto, Ai Nakaoka, Takeaki Ishihara, Daisuke Miyawaki, Hitoshi Ashida, Ryohei Sasaki

**Affiliations:** 1 Division of Radiation Oncology, Graduate School of Medicine, Kobe University, Kobe 650-0017, Japan; shimizuy@med.kobe-u.ac.jp (Y.S.); shunta@omu.ac.jp (S.H.); nmukumot@med.kobe-u.ac.jp (N.M.); anakaoka@east.ncc.go.jp (A.N.); take3036@med.kobe-u.ac.jp (T.I.); miyawaki@med.kobe-u.ac.jp (D.M.); 2 Radiological Division, Osaka Metropolitan University Hospital, Osaka 545-8586, Japan; 3 Biochemistry Department, Faculty of Veterinary Medicine, South Valley University, Qena 83523, Egypt; 4 Division of Gastroenterology, Department of Internal Medicine, Graduate School of Medicine, Kobe University, Kobe 650-0017, Japan; nhoshi@med.kobe-u.ac.jp; 5 Department of Agrobioscience, Graduate School of Agricultural Science, Kobe University, Kobe 657-0013, Japan; yoko.y@crystal.kobe-u.ac.jp (Y.Y.); ashida@kobe-u.ac.jp (H.A.); 6 Division of Dermatology, Department of Internal Related, Graduate School of Medicine, Kobe University, Kobe 650-0017, Japan; fuku@med.kobe-u.ac.jp; 7 Faculty of Food Science and Nutrition, Mukogawa Women’s University, Nishinomiya 663-8558, Japan

**Keywords:** colorectal cancer, Tamba, proanthocyanidins, black soybeans, colorectal prevention

## Abstract

Background: Proanthocyanidins (PACs) from black soybean seed coat have antioxidant and anti-tumorigenic properties. We investigated the anti-tumor properties and mechanisms of action of PACs on colorectal cancer (CRC). Methods: We fed the APC^min/+^ mice, which are highly susceptible to spontaneous intestinal adenoma formation, diets supplemented with or without PACs for 7 weeks and assessed adverse effects, the number and size of intestinal polyps, and the expression of pro- and anti-proliferative proteins in the intestine. The mouse gut microbiome composition was analyzed, and the concentrations of gut short-chain fatty acids (SCFAs) were quantified. We also compared CRC incidence in Tamba in Japan, where black soybean is consumed frequently, with that in the rest of Japan. Results: The number and size of intestinal polyps notably decreased in the PAC-fed mice. Compared with control mice, the PAC-fed mice showed lower expression of proliferation markers proliferating cell nuclear antigen and β catenin and a higher expression of the anti-inflammatory protein oligomeric mucus gel-forming. PAC supplementation increased the prevalence and concentrations of beneficial gut microbes and SCFAs, respectively. Conclusions: Diet supplemented with black soybean-derived PACs could prevent CRC development in mice through gut microbiome remodeling. Regions consuming black soybeans have low CRC incidence. Notably, the incidence of CRC, breast cancer, and liver cancer was significantly lower in Tamba than in the rest of Hyogo Prefecture or Japan. Future studies should delineate the mechanisms underlying the CRC-protective effects of PACs. Nevertheless, our results demonstrate the potential of including PACs in dietary recommendations for cancer prevention.

## Simple Summary

This study explores the colorectal cancer (CRC)-preventive potential of proanthocyanidins (PACs) extracted from black soybean coats. A PAC-supplemented diet decreased intestinal polyp development in a mouse model. It also increased the prevalence of beneficial gut microbiota and the concentrations of beneficial short-chain fatty acids, which might have contributed to the reduction in polyp formation. Interestingly, our analysis revealed a low CRC incidence in a population that consumes black soybeans frequently.

## 1. Introduction

Colorectal cancer (CRC) is a leading cause of cancer-related mortality both in Japan and globally [1,2]. Extensive epidemiological and experimental evidence suggests that a Western-style diet, characterized by high fat and red meat intake and low fiber and vegetable consumption, elevates the risk of CRC [3,4]. Identifying dietary components that prevent CRC has been a significant research focus in recent years. Dietary phytochemicals comprising a diverse array of biologically active compounds have garnered considerable attention from both the scientific community and the general public owing to their potential to suppress CRC [5,6].

Natural products have been recognized as a source of chemopreventive agents for many years [7]. Plant-based bioactive compounds have several health benefits. Many in vitro and epidemiological studies have shown that plant-derived compounds, such as polyphenols, flavonoids, plant sterols, salicylates, and glucosinolates, have promising chemopreventive potential [8,9]. Extensive research has explored the effects of both pure proanthocyanidins (PACs) and PAC-rich extracts on overall health. As antioxidants, PACs reduce the production of reactive oxygen species (ROS), which induce DNA damage and promote carcinogenesis [10]. These findings suggest that the long-term intake of PACs has the potential to reduce tumorigenesis in the intestine. However, studies on the CRC-preventive potential of PACs derived from black soybeans are lacking.

Black soybean is a PAC-rich soybean that is widely used as a nutritionally rich food in Asia. Black soybeans, which are different from yellow soybean seed coats, are abundant in polyphenols [11,12]. Among these polyphenols, anthocyanins and PACs have been reported to play important roles in the prevention of oxidative damage and to have a broad range of effects, including anti-atherosclerotic, anti-inflammatory, and anti-carcinogenic properties [13,14]. Despite studies on the nutritional benefits of black soybeans, no study has explored the CRC-protective benefits of consuming black soybeans in the Japanese population.

APC^min/+^ mice mimic human intestinal carcinogenesis and are a well-established model for studying malignant transformation in intestinal tumorigenesis [15]. They carry a germline nonsense mutation at codon 850 of the mouse homolog of the human adenomatous polyposis coli (APC) gene and spontaneously develop multiple polyps in the small and large intestines at 10–12 weeks of age [16]. APC is a tumor-suppressor gene whose mutation has been directly implicated in the development of human familial adenomatous polyposis (FAP) and sporadic CRC [17,18]. Disordered APC protein leads to decreased β-catenin degradation concomitant with the activation of the Wnt pathway [19,20]. β-catenin accumulates in the nucleus and binds to transcription factors belonging to the lymphoid-enhancing factor (LEF-1) family, which augments the transcriptional level of target genes, including the cyclin D1 gene [21]. Owing to these reasons, the APC^min/+^ mouse model is an ideal experimental model for studying the effect of CRC prevention strategies.

The gut microbiome plays a crucial role in the degradation of dietary fibers and bioactive compounds, facilitating the production of short-chain fatty acids (SCFAs), including acetate, propionate, and butyrate, all of which significantly influence the host’s health. These SCFAs are synthesized through the fermentation of indigestible carbohydrates derived from plant fibers. They contribute to the maintenance of gut health, modulation of the immune system, and regulation of metabolic processes, such as lipid and glucose metabolism. SCFAs, particularly butyrate, are recognized for their potential to enhance intestinal barrier integrity, reduce inflammation, and offer protective effects against conditions such as colorectal cancer [22].

In the present study, we used the APC^min/+^ mouse model to investigate the inhibitory activity of PACs against intestinal tumorigenesis and the possible mechanisms involved in its inhibitory action. We hypothesized that supplementing the diet with PACs might suppress carcinogenesis in APC^min/+^ mice. We further compared the prevalence of CRC in a population that consumes black soybean, a significant source of PACs, to that in the rest of Japan. Our results showed that PAC supplementation significantly reduced polyp development in APC^min/+^ mice and might be contributing to the lower CRC incidence in Tamba, where people consume black soybeans throughout the year.

## 2. Materials and Methods

### 2.1. Animals

APC^min/+^ male mice with a C57BL/6 genetic background were obtained from Jackson Laboratories (ME, USA) and bred with wild-type C57BL/6 mice by in vitro fertilization to accelerate breeding efficiency. They were housed under specific pathogen-free conditions in the Animal Facility at Kobe University Graduate School of Medicine. The animals were maintained in 12 h light/dark cycles with free access to water and food (AIN-76A powder diet from CLEA Japan, Inc., Tokyo, Japan). The APC^min/+^ genotype was confirmed by polymerase chain reaction (PCR) analysis using DNA extracted from the severed tail tip [23]. The APC gene was amplified in the presence of dNTPs using the following primer pairs: Apc-mutant forward (TTCTGAGAAAGACAGAAGTTA) and Apc-common reverse (TTCCACTTTGGCATAAGGC); Apc-wild type forward (GCCATCCCTTCACGTTAG), and Apc-common reverse (TTCCACTTTGGCATAAGGC). APC^min/+^ mice were used to investigate the inhibitory effects of PAC supplementation on intestinal tumorigenesis. PCR was carried out using the Thermal Cycler Dice Real Time System III from TaKaRa, Osaka, Japan, and the products were generated with LA Taq from TaKaRa, Osaka, Japan. Specifications: 94 °C for 180 s; 94 °C for 40 s, 65 °C for 40 s, 72 °C for 300 s (35 rounds); 72 °C for 300 s. The PCR bands were visualized using UV irradiator (DT-20MP, ATTA, Japan) to examine a 600 bp wild-type band and a 340 bp APC^Min^ band. The experimental procedures were conducted in accordance with the guidelines for the care and use of laboratory animals. All animal experiments were approved by the Kobe University Ethics Review Committee for Animal Research (P200508).

### 2.2. Diet Supplementation and Administration of PACs

Five-week-old wild-type and APC^min/+^ mice were randomly divided into three subgroups of five animals each. The mice were fed AIN-76A or AIN-76A supplemented with either 0.05% or 0.5% PACs, a component of the black soybean seed coat. All mice received the diet ad libitum for 7 weeks. The AIN-76A diet was prepared by the Faculty of Agriculture, Kobe University. Black soybean seed coat extract (Chrono-Care, Fujicco Co., Ltd., Kobe, Japan) was prepared as previously described [24]. It contains abundant polyphenols such as 9.2% cyanidin 3-glucoside, 6.2% epicatechin, and 39.8% PACs [25].

### 2.3. Immunohistochemical Evaluation and Enumeration of the Polyps

The intestines were collected after mice euthanasia, sliced longitudinally, rinsed with saline, and mounted onto slides. The intestinal polyps were counted, and their sizes were measured under a dissecting microscope. Intestinal polyps were categorized by size (diameter) as 1–2, 2–3, and >3 mm. Subsequently, parts of the small and colonic intestines were snap-frozen in liquid nitrogen for Western blot analysis. Other parts of the intestine were placed in 10% phosphate-buffered formalin for histopathological and immunohistochemical analysis. The samples were dehydrated and embedded in paraffin, as described previously [26].

In further experiment, the intestines were collected, spread onto filter paper, opened longitudinally with fine scissors, and cleaned with sterile PBS. The small intestine was divided into three equal parts (proximal, middle, and distal), and the colon consisted of the large intestine and rectum. All intestinal polyps were fixed with 10% formalin and stained with methylene blue. Images were captured using a Video-Zoom-microscope XV-440 (Wraymer, Osaka, Japan), taken under 7× magnification, and counted using Spectoman for Mac (Wraymer). Mean ± SE values were obtained from the evaluation of multiple fields in each group. For each animal, ten representative fields were counted at 400× magnification, and the data represent the results from five mice in each group.

### 2.4. Immunohistochemistry

The intestines were fixed in 10% phosphate-buffered formalin for 10 h at 4 °C, dehydrated in ascending concentrations of ethanol, cleared with xylene, and embedded in PolyFin (Triangle Biomedical Sciences, Durham, NC, USA). Paraffin-embedded tissue blocks were cut using a rotary microtome into 4 μm sections and processed for immunohistochemical staining. Briefly, sections were deparaffinized, rehydrated, and treated with 0.01 M sodium citrate buffer (pH 6.0) in a microwave for 30 min at full power for antigen retrieval. The endogenous peroxidase activity was quenched by immersion in 3% hydrogen peroxide for 5 min at room temperature. The sections were then incubated either with mouse monoclonal anti-proliferating cell nuclear antigen (PCNA) antibody (1:400 dilution; Dako, Carpinteria, CA, USA), rabbit polyclonal anti–β-catenin (1:100 dilution; Santa Cruz Biotechnology, Inc., Santa Cruz, CA, USA), or rabbit polyclonal anti–MUC2 (1:1500 dilution; Invitrogen, Etobicoke, ON, Canada) in PBS for 2 h at room temperature in a humidity-controlled chamber followed by overnight incubation at 4 °C. Sections stained with N-Universal Negative Control mouse or rabbit antibody (Dako, Troy, MI, USA) under identical conditions were used as negative controls. The sections were then incubated with the appropriate biotinylated secondary antibody for 1 h at room temperature, followed by a 30 min incubation with HRP-conjugated streptavidin. Color development was performed by incubating the sections with 3,3′-diaminobenzidine for 10 min at room temperature. The sections were counterstained with Harris hematoxylin, dehydrated, and mounted.

### 2.5. Gut Microbiota Analysis

Mice fecal samples from 13-week-old offspring were collected and maintained until use at −30 °C. DNA from stool samples was extracted using the QIAamp R Fast DNA Stool Mini Kit (QIAamp, Hamburg, Mannheim, Germany). The 16 s V3–V4 hypervariable region was targeted for sequencing amplification. Fecal microbiota composition was detected by 16 S rRNA sequencing (Realbio Genomics Institute, Shanghai, China) using an Illumina HiSeq platform (Illumina, San Diego, CA, USA). The optimizing sequences were mapped into operational taxonomic units (OTUs) and picked at 97% similarity in Mothur (version v.1.30.1). The data were analyzed using R Cluster analysis (version 3.5.1) and QIIME pipeline (Version 1.9.1). The resulting matrix of distances was determined using principal component analysis (PCA) between the different indicated groups. The abundance and diversity were estimated by the Shannon and Simpson indices. Analyses of the intestinal SCFA concentrations were outsourced to Techno Suruga Laboratory Co., Ltd. (Shizuoka, Japan).

### 2.6. Short-Chain Fatty Acids Analysis

Fecal samples were gathered in a test tube and stored at −20 °C. The SCFA concentrations were analyzed by Techno Suruga Laboratory Co., Ltd. (Shizuoka, Japan). The feces were placed in 2.0 mL tubes with zirconia beads and mixed in MilliQ water. The specimens were warmed at 85 °C for 15 min, agitated at 5 m/s for 45 s with FastPrep-24 5 G (MP Biomedicals, Irvine, CA, USA), and spun at 15,350× *g* for 10 min. The supernatant was filtered using 0.2 μm filter. The concentrations of SCFAs, including succinic acid, lactic acid, formic acid, acetic acid, propionic acid, and butyric acid, were analyzed using a high-performance liquid chromatography (HPLC) system equipped with an Aminex HPX-87H column (Bio-Rad Laboratories, Hercules, CA, USA) and a RID-10A refractive index detector (Shimadzu, Kyoto, Japan).

### 2.7. Evaluation of the Incidence of Cancers in Tamba, Hyogo Prefecture, Japan

Japan has 47 prefectures, each of which conducts a cancer registry survey every year. The incidence of six major cancers (colorectal cancer, breast cancer, liver cancer, lung cancer, gastric cancer, and uterine cancer) from 2011 to 2015 in Hyogo Prefecture, which is located in western Japan, was investigated using the population-based, age-standardized Cancer Registry of Japan [1]. All registry data on six cancers from ten areas (Kobe, South-Hanshin, North-Hanshin, East-Harima, North-Harima, Middle-Harima, West-Harima, Tajima, Tamba, and Awaji) of Hyogo Prefecture were independently registered. Notably, the incidence of CRC, breast cancer, and liver cancer was significantly lower in Tamba, Hyogo Prefecture, Japan, than in the rest of Hyogo Prefecture or Japan.

### 2.8. Statistical Analysis

The mean ± standard deviation is presented. Data were analyzed statistically by one-way ANOVA with Tukey’s multiple comparisons. GraphPad Prism 9.0 (GraphPad Software, La Jolla, CA, USA) was used to analyze all the data. Statistical significance was established at * *p* < 0.05, ** *p* < 0.01, and *** *p* < 0.001.

## 3. Results

### 3.1. PAC Supplementation Attenuated Polyp Formation in APC^min/+^ Mice

**Figure 1:**
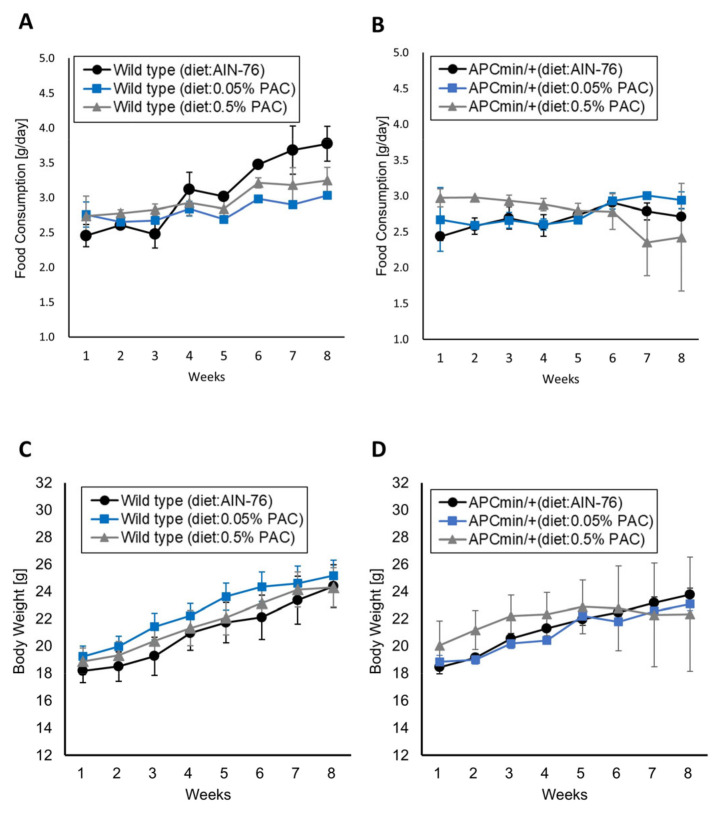
Adverse effects of dietary PACs on mice body. (**A**,**B**) Food consumption in different indicated groups. (**C**,**D**) Body weight in different indicated groups. Data are shown as the mean ± standard deviation.

All mice were monitored regularly to determine the effect of dietary black soybeans on body weight. First, we evaluated the effects of PACs on food consumption and body weight. In the wild-type mice, the food consumption of the mice fed a normal diet was slightly higher than that of mice fed a diet containing 0.05% or 0.5% PACs (Figure 1A,B). However, the body weights of the wild-type mice in the different groups were similar. No differences in food consumption or body weight were found between the different groups of APC^min/+^ mice (Figure 1C,D).

**Figure 2:**
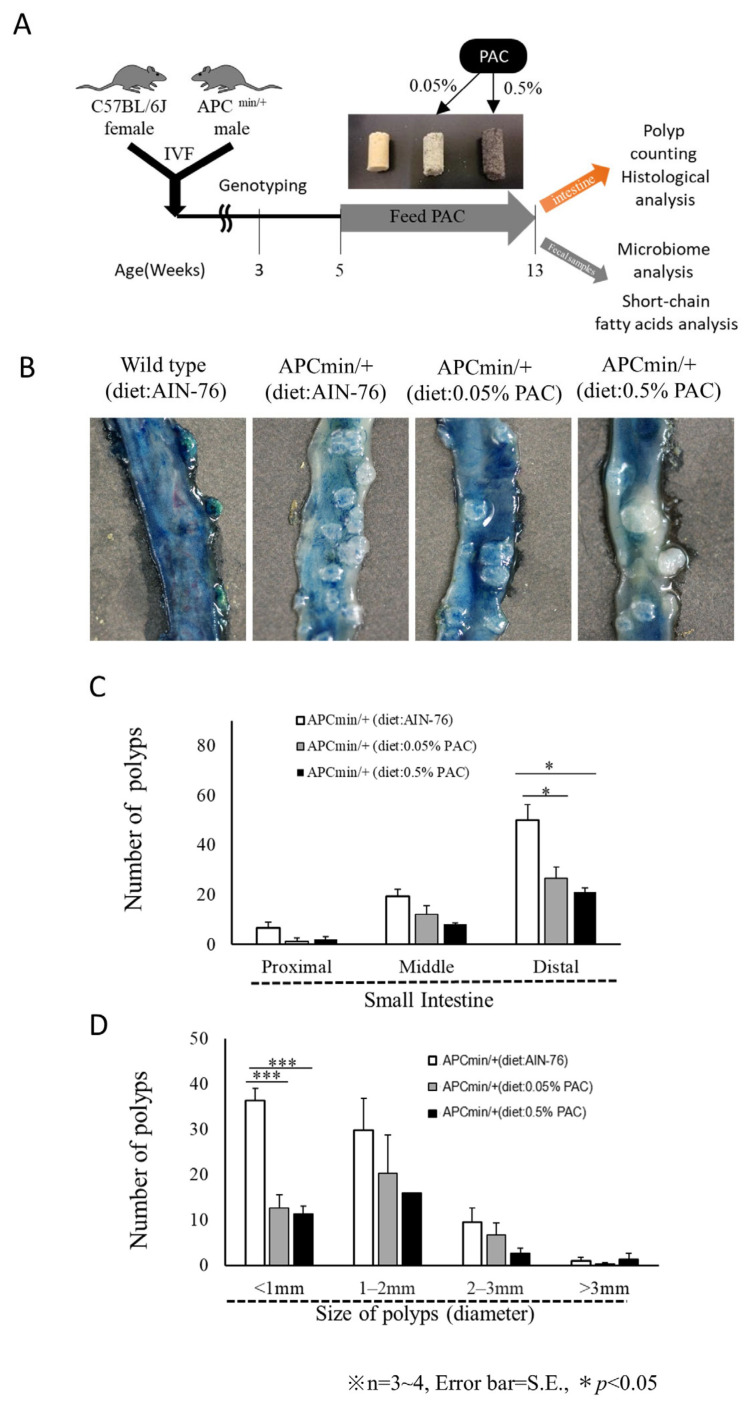
Gross examination of the intestinal tumors. (**A**) Schematic of the experimental plan. (**B**) Methylene blue staining of the small intestines from wild-type and APC^min/+^ mice fed with AIN-76 supplemented with or without PAC supplementation. (**C**) Number of polyps in different intestinal regions in the APC^min/+^ mice. (**D**) Number of polyps grouped based on the polyp diameter in different intestinal regions in the APC^min/+^ mice. Data represent the mean ± standard deviation. One-way ANOVA with Tukey’s post hoc test was used * *p* < 0.05 and *** *p* < 0.001.

The intestinal tracts of the mice fed AIN-76A or AIN-76A supplemented with either 0.05% or 0.5% PACs and received the diet ad libitum for 7 weeks were collected at 12 weeks of age for further analyses (Figure 2A). The intestinal tissues from different groups were stained with methylene blue and hematoxylin and eosin. The APC^min/+^ mice fed a normal diet spontaneously developed intestinal polyps, as expected (Figure 2B). Interestingly, the APC^min/+^ mice fed the PAC-containing diet (0.05% or 0.5%) had significantly fewer intestinal polyps (Figure 2B). The number of polyps varied in different intestinal regions of the intestine. The distal portion most frequently developed polyps, followed by the middle portion. PAC supplementation inhibited tumor growth at all regions in the intestine (Figure 2C). The distal portion showed marked reductions in the number and size of polyps in the PAC-fed APC^min/+^ mice. PAC-fed mice had significantly fewer polyps that were lower than 1 mm in diameter (*p* < 0.001) (Figure 2D).

**Figure 3:**
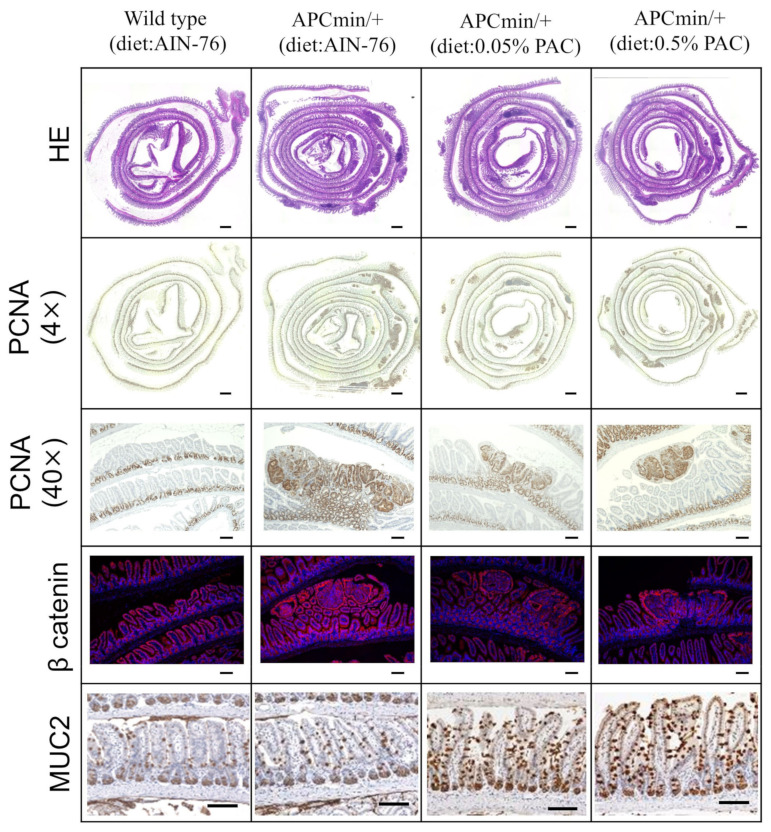
Microscopic examination of H&E staining, PCNA expression, β-catenin expression, and MUC2 expression in the experimental groups. Scale bar: for HE and PCNA (×4) is 500 µm and for PCNA (×40), β-catenin, and MUC2 is 100 µm.

The characteristics of the tumorous lesions were evaluated by staining with hematoxylin and eosin and periodic acid-Schiff reaction. Tumorous lesions were clearly reduced in the APC^min/+^ mice fed the PAC-containing diet (0.05% or 0.5%). Supplementing the diet with 0.5% PACs showed the maximum suppression of gut polyp formation in the APC^min/+^ mice. Furthermore, the protein expression levels of PCNA and β catenin were decreased in PAC-fed APC^min/+^ mice, with the higher concentration of PACs showing the lowest PCNA and β-catenin expression. By contrast, MUC2 was overexpressed in the PAC-fed mice compared with the AIN-76-fed APC^min/+^ mice (Figure 3).

### 3.2. PAC Supplementation Promoted Gut Microbiota Remodeling in Mice

**Figure 4:**
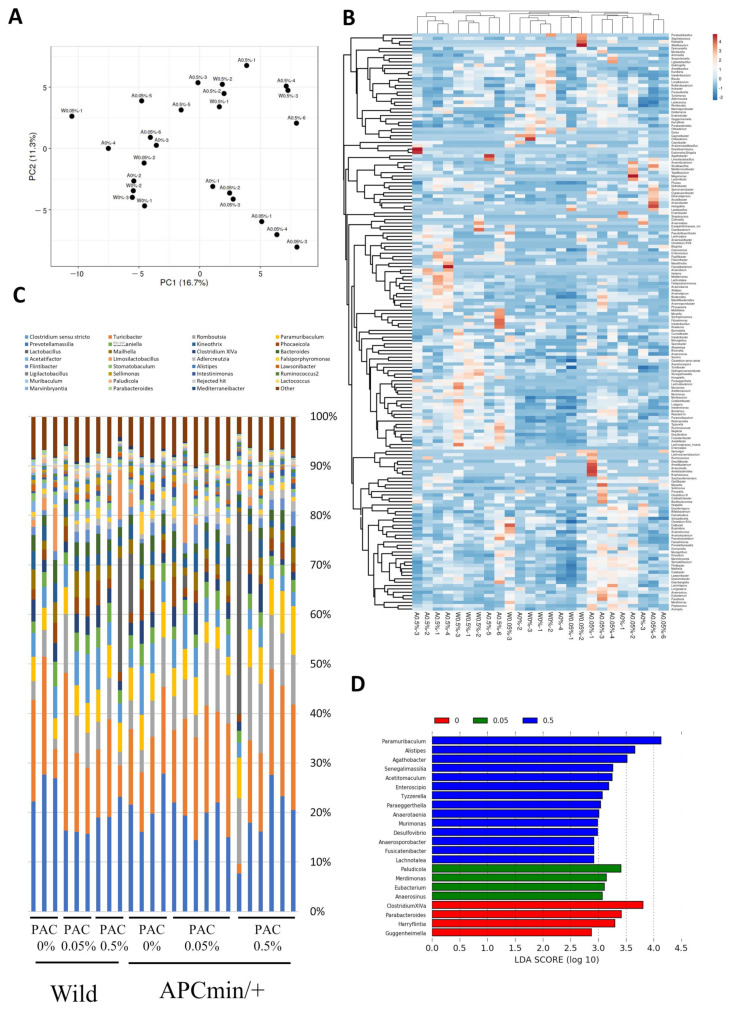
PAC supplementation remodeled the gut microbiome of APC^min/+^ mice. (**A**) PCA plot of microbiome samples. The first component (PC1) explained 16.7% of the total variance, and PC2 accounted for 11.3%. The same labels indicate samples that originated from the same individual. (**B**) Hierarchical clustering of the gut microbiome of the different groups. (**C**) Distribution of the gut microbiome in the different groups. (**D**) Biomarkers found by LEfSe ranked according to their effect size and associating them with the class with the highest median.

The gut microbiota in the wild-type and APC^min/+^ mice fed on the AIN-76A diet with or without PAC supplementation was evaluated. PCA was performed, where the first component (PC1) recorded 16.7% of the total variance, while PC2 explained 11.3% (Figure 4A). Gut microbiota hierarchical clustering showed around 200 different gut microbiota components with varying distributions between the experimental groups (Figure 4B). We further analyzed the top 32 components in the different groups (Figure 4C). A linear discriminant analysis was conducted where the biomarkers found by LEfSe ranked the microbiota according to their effect size and incorporation with the class with the highest median. We recorded 14 gut genera enriched in the APC^min/+^ mice fed on 0.5% PACs, 4 enriched in mice fed on 0.05% PACs, and 4 genera enriched in mice fed on the supplemented AIN-76A diet (Figure 4D).

**Figure 5:**
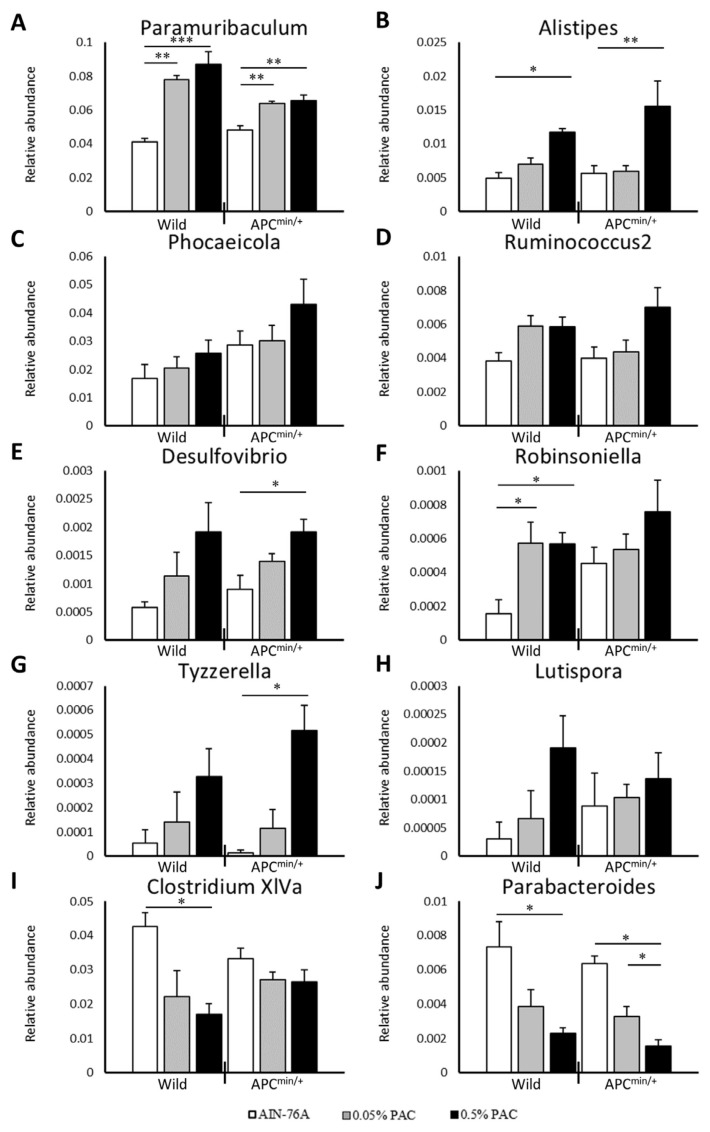
PAC supplementation increased the number of beneficial gut microbiota. Relative abundance of gut microbiota *Paramuribaculum* (**A**), *Alistipes* (**B**), *Phocaeicola* (**C**), *Ruminococcus* (**D**), *Desulfovibrio* (**E**), *Robinsoniella* (**F**), *Tyzzerella* (**G**), *Lutispora* (**H**), *Clostridium* XIVa (**I**), and *Parabacteroides* (**J**) is shown. Data represent mean ± standard deviation. One-way ANOVA with Tukey’s post hoc test was used. * *p* < 0.05, ** *p* < 0.01 and *** *p* < 0.001.

We analyzed the relative abundance of the gut microbiota in the wild-type and APC^min/+^ groups fed different diets. The relative abundance of beneficial genera *Paramuribaculum*, *Alistipes*, *Desulfovibrio*, and *Tyzzerella* significantly increased in the APC^min/+^ mice fed 0.5% PACs compared with the mice fed the unsupplemented AIN-76A diet (*p* < 0.01, *p* < 0.01, *p* < 0.05, and *p* < 0.05; respectively), whereas no significant differences were found between the mice fed 0.05% PACs and the mice fed the unsupplemented AIN-76A (Figure 5A,B,E,G). The relative abundance of *Parabacteroides* significantly decreased in the mice fed the 0.5% PAC-supplemented diet compared with the mice fed the unsupplemented AIN-76A or 0.05% PAC-supplemented diet (*p* < 0.05) (Figure 5J). Supplementing the diet with 0.5% PACs noticeably affected the populations of *Paramuribaculum* and *Parabacteriodes* (*p* < 0.01 and *p* < 0.01, respectively) (Figure 5A,J) but did not affect the populations of *Phocaeicola*, *Ruminococcus*, *Robinsoniella*, *Lutispora*, and *Clostridium* XIVa in the mice fed the 0.5% or 0.05% PAC-supplemented diet compared with the mice fed the unsupplemented AIN-76A diet (Figure 5C,D,F,H,I).

**Figure 6:**
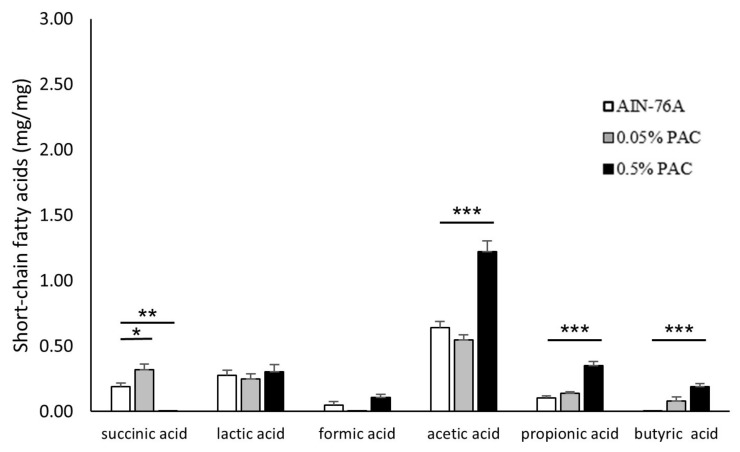
PAC-rich diet increased the concentrations of beneficial gut SCFAs in the APC^min/+^ mice. The concentrations of succinic, lactic, formic, acetic, propionic, and butyric acids were measured in the experimental groups. Data represent mean ± standard deviation. One-way ANOVA with Tukey’s post hoc test was used. * *p* < 0.05, ** *p* < 0.01 and *** *p* < 0.001.

Finally, we explored the production of SCFAs from the intestinal microbiota (Figure 6). The levels of acetic, propionic, and butyric acids markedly increased in the PAC-fed mice compared with the control mice. The 0.5% PAC group showed a maximum production of these fatty acids, which might indicate the beneficial effect of these SCFA-producing microbiota in suppressing intestinal tumors. Although the concentration of succinic acid was elevated in the 0.05% PAC group, it significantly decreased in the 0.5% PAC group compared with the control group. No change in the production of formic and lactic acid was recorded in the mice fed the 0.5% or 0.05% PAC-supplemented diet compared with the mice fed the unsupplemented AIN-76A diet.

### 3.3. Tamba Region in Japan, Where Black Soybeans Are Consumed More Frequently, Had a Lower Incidence of CRC

**Figure 7:**
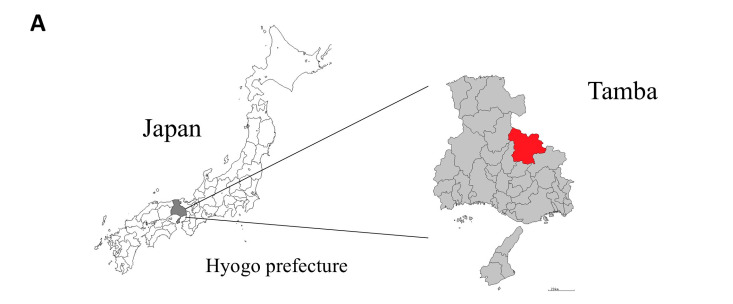

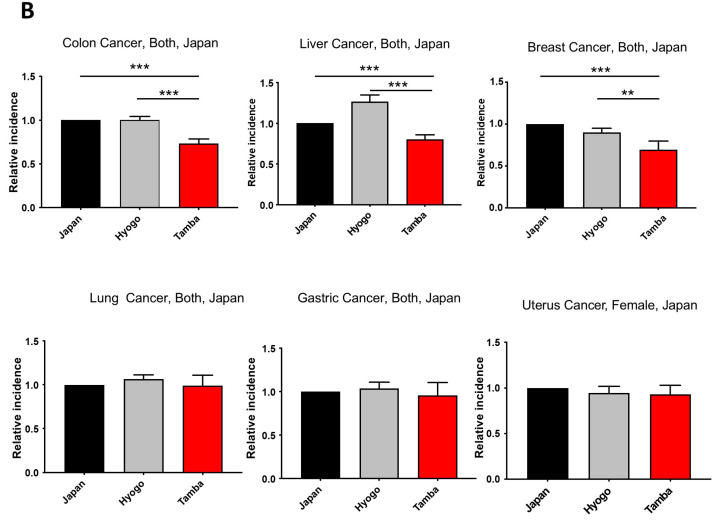
Relative cancer incidence in Tamba compared with the rest of Hyogo Prefecture or Japan. (**A**) Map showing the location of Tamba in Hyogo Prefecture, Japan. (**B**) Relative incidence of different cancers in Tamba, rest of Hyogo (excluded Tamba), and Japan. Data are shown as the mean ± standard deviation. One-way ANOVA with Tukey’s post hoc test was used. ** *p* < 0.01 and *** *p* < 0.001.

Considering that our in vivo results suggested that PAC supplementation might contribute to a CRC-protective effect, we compared the incidence of different cancers in Tamba, where people consume black soybeans more frequently and throughout the year, with that in the rest of Hyogo Prefecture or Japan. Interestingly, Tamba (Figure 7A) had a significantly lower incidence of colon cancer than the rest of Hyogo Prefecture or Japan (*p* < 0.001, Figure 7B). Similarly, the incidence rates of liver and breast cancers were also significantly lower in Tamba than in the rest of Hyogo Prefecture or Japan. On the other hand, the incidence rates of lung cancer, gastric cancer, and uterine cancer were similar to those of Hyogo and all over Japan. These suggested a positive impact of black soybean consumption on CRC incidence.

## 4. Discussion

APC^min/+^ mice are heterozygous for a truncating mutation in the APC tumor-suppressor gene and provide a model of FAP and sporadic colorectal cancers [16]. This mouse model is unique in that tumors appear spontaneously in the gastrointestinal tract rather than being induced by a carcinogen. It is particularly useful for studying the effect of cancer prevention strategies and therapies that target the early stages of carcinogenesis.

Our results demonstrated that supplementing the diet with 0.5% PACs significantly suppressed intestinal tumorigenesis in the APC^min/+^ mice. In particular, we observed a significant reduction in small tumors and tumors in the distal region of the small intestine. Moreover, this cancer-preventive effect was achieved without any significant adverse effect, such as weight loss or changes in food consumption.

APC^min/+^ mice form a large number of polyps, which are precursors of CRC, but rarely develop CRC [18]. Therefore, one of the limitations of this model is that it does not directly examine the development of CRC. However, since mutations in APC are involved in the development of FAP and sporadic CRC, it is considered an ideal experimental model for the analysis and prevention of human CRC.

Our results suggest that oral administration of PAC extracted from black soybean seed coat contributes to a decrease in polyp formation in APC^min/+^ mice. Both gross anatomic and microscopic evaluations confirmed the marked reduction in the polyp number and size. Furthermore, PAC supplementation decreased the expression of proliferation markers in the intestinal polyps and enhanced the production of mucins in the intestinal mucosa in a concentration-dependent manner. Mucins are crucial elements of the mucus that serve a double purpose, as they protect against pathogens and offer nutrients for microorganisms. Nonetheless, in conditions such as inflammatory bowel diseases, CRC, and metabolic disorders, detrimental bacteria produce metabolites that reduce the thickness of the intestinal mucus layer [27].

We speculated that PACs inhibit intestinal malignancies by modulating the gut microbiota populations. Our microbiome analysis showed variations in some gut microbiota in the APC^min/+^ mice fed a diet with or without PAC supplementation. The prevalence of beneficial microbiota, such as *Paramuribaculum*, *Alistipes*, *Desulfovibrio*, and *Tyzzerella*, increased in the APC^min/+^ mice fed a PAC-supplemented diet. In contrast, the prevalence of harmful microbiota, *Parabacteroides*, was significantly reduced in the mice fed a PAC-supplemented diet. These data indicate the major role of microbiota in intestinal tumor suppression.

The microbiota have a crucial impact on the management of CRC by affecting treatment outcomes and patient well-being. Having a healthy gut microbiome can boost the immune system, which helps activate immune cells that fight tumors and make treatments like immunotherapy more successful. For example, certain microbiota in the gut can generate SCFAs, which have anti-inflammatory properties and may hinder tumor advancement [28]. Probiotics and prebiotics can help restore the balance of microbes, reducing dysbiosis seen in many patients with CRC [29]. Additionally, recent research has indicated that customizing treatment plans according to each person’s microbiota characteristics may enhance therapeutic interventions, ultimately resulting in better results for individuals with CRC [30].

Interestingly, our SFCA analysis showed that the levels of acetic, propionic, and butyric acids significantly increased in the APC^min/+^ mice fed a 0.5% PAC-supplemented diet. We speculate that these fatty acids were produced by the beneficial microbiota that were identified in our microbiome analysis, which might have a direct or indirect role in suppressing the CRC. Our results are in line with previous studies that have demonstrated the anti-tumor activity of SCFAs produced by the intestinal microbiome [31,32,33,34,35,36]. Butyric acid, isobutyric acid, and acetic acid inhibit the growth of human CRC cells, with butyric acid showing the strongest inhibition [31,37]. Additionally, butyrate can pass through the intestinal epithelium and reach the lamina propria, directly contributing to the mucosal immune response [38]. These findings indicate the major role of microbiota in ameliorating colorectal cancer therapy.

Considering that our in vivo results suggested the CRC-protective effect of black soybean-derived PACs, we decided to determine the effect of regular black soybean intake on CRC incidence. People in Tamba in Hyogo Prefecture consume black soybeans frequently and throughout the year. Therefore, the cancer incidence in Tamba was compared with that in the rest of Hyogo Prefecture or Japan. In line with the results from our animal study, Tamba showed a significantly lower incidence of colon, breast, and liver cancer. However, no differences were observed in the incidence rates of lung, gastric, or uterine cancers. We speculate that the increased consumption of black soybeans might have contributed to the lower incidence rate of colon cancers. However, we have not accounted for other confounding and risk factors that contribute to CRC pathogenesis. Thus, future studies should consider other confounding factors, including environmental and germline genetic factors, and determine the correlation between black soybean consumption and CRC pathogenesis. The PACs from black soybean coat might be contributing to a different gut microbiome architecture in the people from Tamba, rendering a protective effect on CRC development. However, further detailed studies on the dietary habits, gut microbiome, and CRC incidence rates of people in Tamba should be conducted to confirm this. The justification for this is as follows. Data of each area including Tamba in Hyogo were assessed using the population-based cancer registry of Hyogo Prefecture, whereas data of the Hyogo Prefecture and whole of Japan were obtained from the nationwide population-based cancer registry of Japan [1].

In the human epidemiological investigation, the participants were people who may eat black soybeans and their products often and throughout the year in Tamba. Different from soybeans, which primarily contain isoflavones in the interior, black beans are rich in polyphenols in the seed coat and isoflavones in the interior. Therefore, black soybeans may have a stronger tumor-suppressive effect than soybeans. Previous studies reported that people in Tamba consume black soybean throughout the year in various recipes (Edamame, Kuromame, miso soup, etc.). However, to the best of our knowledge, this study is the first to demonstrate that the black soybean consumption in Tamba is much higher than that in the rest of Hyogo Prefecture. Colon, breast, and liver cancers have been discussed as diet-related diseases, whereas lung, gastric, and uterine cancers have not. Therefore, we speculated that the large consumption of black soybean in Tamba contributes to the low incidence of colon, breast, and liver cancers. Nevertheless, further investigation is needed for confirmation.

The relationship between cancer risk and food consumption remains controversial. Consumption of soy-based foods has been associated with a decreased incidence of many cancers, mainly breast cancer [39]. Wang et al. [40] demonstrated that high consumption of total soy products, especially soymilk and tofu, is associated with low cancer risk. A case–control study nested within the European Prospective Investigation into Cancer and Nutrition study assessed the relationship between pre-diagnostic plasma polyphenols and colon cancer risk [41]. This study demonstrated that the concentration of equol, which is metabolized from soy-based foods, is inversely associated with colon cancer risk. Regarding liver cancer, not only foods, but also hepatitis virus B or C, influence the incidence of hepatocellular carcinoma (HCC); however, the relationship between liver cancer and isoflavones remains controversial. Kurahashi et al. [42] reported that isoflavone consumption may be associated with an increased risk of HCC in women. Sharp et al. [43] reported a statistically significant interaction between sex and HCV, with the risk of HCC being substantially higher in women than in men, and concluded that the consumption of soy-based foods may reduce HCC risk. To the best of our knowledge, no reports have demonstrated a relationship between black soybean and bin incidence in liver cancer.

This study has some limitations. Considering that the cancer registry data of Hyogo Prefecture before 2011 were primitive, we could not adopt them for the analyses. In 2011, the incidence of colon or breast cancers in Tamba was lower than that in the rest of Hyogo Prefecture. The present study was initiated and used data obtained within 2011–2015. Results indicated that the incidence of colon, breast, and liver cancers was lower in Tamba than in the rest of Hyogo Prefecture and the whole of Japan.

## 5. Conclusions

Our results suggest that chemoprevention using dietary PACs from black soybean coat extract is a promising approach for preventing colon cancer in high-risk individuals, such as FAP carriers. Additional preclinical research is required to assess the preventive effects of PACs extracted from the coat of black soybeans in FAP models and conduct clinical trials in at-risk populations. Elucidating the mechanisms underlying microbiome modulation and SCFA production will be helpful to understand how PACs participate in colon cancer prevention and suggest possible dietary approaches.

## Data Availability

Raw sequencing data have been uploaded to NCBI Sequence Read Archive (SRA) under BioProject accession number PRJNA1182577.
